# Subcutaneous, Oral, and Intranasal Immunization of BALB/c Mice with *Leishmania infantum* K39 Antigen Induces Non-Protective Humoral Immune Response

**DOI:** 10.3390/tropicalmed8090444

**Published:** 2023-09-12

**Authors:** Bruno Bezerra da Silva, Amauri Barbosa da Silva Junior, Lucelina da Silva Araújo, Eduarda Nattaly Ferreira Nobre Santos, Ana Cláudia Marinho da Silva, Eridan Orlando Pereira Tramontina Florean, Maurício Fraga van Tilburg, Maria Izabel Florindo Guedes

**Affiliations:** Laboratory of Biotechnology and Molecular Biology, Northeast Biotechnology Network (RENORBIO), State University of Ceará, Fortaleza 60714903, Brazil

**Keywords:** visceral leishmaniasis, K39, immunization, humoral response, recombinant protein

## Abstract

Visceral leishmaniasis is a high-burden disease caused by parasites of the *Leishmania* genus. The K39 kinesin is a highly antigenic protein of *Leishmania infantum*, but little is known about the immune response elicited by this antigen. We evaluated the humoral immune response of female BALB/c mice (*n* = 6) immunized with the rK39-HFBI construct, formed by the fusion of the K39 antigen to a hydrophobin partner. The rK39-HFBI construct was administered through subcutaneous, oral, and intranasal routes using saponin as an adjuvant. We analyzed the kinetics of IgG, IgG1, and IgG2a production. The groups were then challenged by an intravenous infection with *L. infantum* promastigote cells. The rK39-HFBI antigen-induced high levels of total IgG (*p* < 0.05) in all groups, but only the subcutaneous route was associated with increased production of IgG1 and IgG2a 42 days after immunization (*p* < 0.05), suggesting a potential secondary immune response following the booster dose. There was no reduction in the splenic parasite load; thus, the rK39-HFBI failed to protect the mice against infection under the tested conditions. The results presented here demonstrate that the high antigenicity of the K39 antigen does not contribute to a protective immune response against visceral leishmaniasis.

## 1. Introduction

Leishmaniasis comprises a group of neglected tropical diseases caused by protozoa belonging to the genus *Leishmania* (Trypanosomatida: Trypanosomatidae). Among the 20 or more species that affect humans, *Leishmania infantum* and *L. donovani* are responsible for the most severe form of the disease, known as visceral leishmaniasis [1,2]. These parasites undergo a life cycle involving an insect vector and a mammalian host. The primary reservoir for *L. infantum* is the dog (*Canis lupus familiaris*), which makes visceral leishmaniasis a significant zoonotic concern [3].

In humans, following an incubation period that varies from two to six months, individuals afflicted by *L. infantum* may develop symptoms including prolonged and irregular fevers, weakness, weight loss, hepatomegaly, splenomegaly, anemia or pancytopenia, as well as hypergammaglobulinemia. If left untreated, the condition advances, leading to fatality in 90% of cases [4,5].

In 2018, out of the total number of deaths worldwide due to visceral leishmaniasis (571 deaths), 64.4% (368 deaths) occurred in Brazil. The country bears approximately one-fifth of all global cases and 97.3% of cases in the Americas [6]. The epidemiological data highlights the need for the development of new preventive and therapeutic strategies to eliminate visceral leishmaniasis as a public health issue by 2030, as outlined in the document “A Road Map for Neglected Tropical Diseases 2021–2030” [7,8]. Therefore, a detailed understanding of the complex immune response elicited by these parasites is required [9].

Antibodies generated by B lymphocytes constitute the principal effectors of the humoral adaptive immune response. Nevertheless, in the context of visceral leishmaniasis, the stimulation of these cells can trigger an immunopathological reaction. The hallmark hypergammaglobulinemia associated with this condition arises from an overproduction of immunoglobulins G, some of which possess a low affinity for the pathogen or exhibit self-reactivity. This circumstance can lead to vasculitis initiated by the deposition of immune complexes [10,11,12].

The K39 kinesin is a motor protein found in the cytosol of both promastigote and amastigote forms of the parasite [13]. During natural infections, the pathogen’s destruction through innate immune mechanisms can lead to the host’s initial exposure to intracellular antigens of the protozoa [9]. The K39 antigen comprises a sequence of 39 amino acid residues arranged in tandem within the C-terminal region of this motor protein. The repetitive structure of this antigen renders it a potential immunogen capable of inciting an adaptive humoral response [14].

Since no study has yet characterized the immune response induced by this antigen, this work aimed to evaluate the humoral immune response triggered by the K39 antigen in healthy female BALB/c mice and if this antigen could impact the parasite load in these animals after a challenge with *L. infantum*.

## 2. Materials and Methods

The K39 antigen from *L. infantum* was fused with a hydrophobin tag for purification, and the resulting construct, rK39-HFBI, was transiently expressed in *Nicotiana benthamiana* plants. The expressed construct was semi-purified and used as an immunogen administered via intranasal, oral, and subcutaneous routes, along with *Quillaja saponaria* saponins as the adjuvant. The kinetics of total immunoglobulin G (IgG), IgG1, and IgG2a were determined. The parasite burden in the spleen of challenged animals was also evaluated.

### 2.1. rK39-HFBI Protein Expression and Purification

The processes of gene synthesis, cloning, expression, and purification of the rK39-HFBI construct have been described previously by our group [15]. In summary, the *L. infantum* K39 gene (Accession Number: DQ831678.1) was optimized for expression in *N. benthamiana* and synthesized by BioBasic Inc. (Markham, ON, Canada). The synthesized construct was subcloned into the pCAMGate-ER-HFBI expression vector and subsequently transformed into chemically competent *Agrobacterium tumefaciens* LBA4404 (Invitrogen, Waltham, MA, USA, 18313015). The *A. tumefaciens* culture carrying the K39-HFBI construct was combined with another culture containing a plasmid with the p19 silencing inhibitor. The resulting mixture was infiltrated into the leaves of 6–8 weeks-old *N. benthamiana* plants. After four days, the infiltrated leaves were harvested for total protein extraction, followed by purification using 4% (*v*/*v*) Triton™ X-114 (Sigma, St. Louis, MO, USA, X114). These procedures were also applied to obtain leaves infiltrated solely with p19 (control plants).

### 2.2. Mice Immunization

The amount of antigen for each immunization route was determined in advance. Groups of three female BALB/c mice (6–8 weeks old) received a single dose of the semi-purified rK39-HFBI mixed with a *Quillaja saponaria* saponin extract (Sigma, 47036), used as an adjuvant [16]. For the subcutaneous and oral routes, evaluations were conducted with 10, 20, and 50 micrograms of the K39-HFBI antigen mixed with 16 µg of saponin per animal. Since the intranasal route requires less antigen [17], evaluations were performed at 1, 5, and 10 micrograms of K39-HFBI antigen mixed with 8 µg of saponin for each mouse. For each immunization route, the lowest dose capable of inducing a strong production of total IgG at 14 days post immunization was selected for the immunization assay.

Once the doses of the immunogen for each route have been determined, six female BALB/c mice (6–8 weeks old) were randomly assigned to one of the groups: intranasal, oral, subcutaneous, and control. Initial blood samples were collected from all groups on day 0 to obtain pre-immune sera. Subsequently, the groups intranasal, oral, and subcutaneous were immunized as described below (Table 1). Boosters were given after 21 and 35 days.

The control group did not receive the immunogen but underwent identical procedures until the parasite challenge. Blood collection occurred through retro-orbital bleeding on the 7th, 14th, 21st, 28th, 35th, and 42nd days. Blood was centrifuged at 4000× *g* for 10 min post-clot formation. The resulting sera were individually collected into labeled microtubes and stored at −20 °C for subsequent analysis.

### 2.3. ELISA for Analysis of the Antibody Production Kinetics

The semi-purified rK39-HFBI was diluted into a coating buffer to the concentration of 10 ng/µL. Then, 100 µL of the diluted extract was used to coat each well of the microplates (Sigma, M9410). After overnight coating at 4 °C, the plates were washed with PBS-T (0.05% Tween^®^ 20 (Sigma, P9416)) and blocked with 1% gelatin (Sigma, G6650) for one hour. After blocking, the plates were washed three times with PBS-T and then incubated for another hour with the diluted sera of the immunized mice (1:1000). The serum from each mouse was individually diluted and analyzed and in duplicate. The washing and incubation steps were once again repeated but now using the peroxidase–conjugated anti-mouse IgG (Invitrogen, G21040), anti-mouse IgG1 (Invitrogen, A10551) or anti-mouse IgG2a (Invitrogen, A10685) antibodies (1:5000). Another washing step was performed, and then 100 µL of 1-Step™ Ultra TMB-ELISA Substrate Solution (Thermo Scientific, Waltham, MA, USA) was added to each well. The plates were incubated for 20 min in the dark, and the reaction was stopped by adding 100 µL of 1 N HCl. The absorbance at 450 nm was read using a microplate reader (Synergy™ 2, Biotek, Winooski, VT, USA). Plates coated with proteins from non-infiltrated plants were used as a control.

### 2.4. Leishmania Infantum Infection

The *L. infantum* promastigote cells (strain MHOM/BR/74/PP75, ATCC^®^ 50133™) were grown in Schneider’s Drosophila Medium (Gibco, Grand Island, NY, USA, 21720024) supplemented with 10% (*v*/*v*) of Heat-inactivated Fetal Bovine Serum (Gibco, 10082139), 10% (*v*/*v*) of sterile human urine and 1% (*v*/*v*) of penicillin–streptomycin (10,000 U/mL) (Gibco, 15140163) [18]. The late log culture was collected via centrifugation (1000× *g*; 10 min) and washed with sterile saline. After another centrifugation, the cells were diluted in sterile saline to a final concentration of 1 × 10^8^ cells/mL. One week after the last blood collection, all the groups were challenged by the intravenous administration of 1 × 10^7^ *L. infantum* promastigote cells [19].

### 2.5. Parasite Burden

On the 42nd day after the infection, all the mice were anesthetized and euthanized by cervical dislocation. Each animal had its spleen collected, placed in RNA protective solution (RNAlater, Ambion, Singapore), and stored under refrigeration until DNA extraction. A fragment equivalent to one-quarter of each spleen was disrupted on a bead beater and then submitted to DNA extraction using the Trizol reagent (Invitrogen) following the manufacturer’s protocol. The purified DNA was quantified (Synergy 2, Biotek), and all the samples had a 260/280 absorbance ratio higher than 1.8. Trizol reagent was also used in the DNA extraction protocol to prepare two standard curves for parasite burden normalization and quantification. A standard curve (3 × 10^5^ to 1 × 10^2^) was prepared for normalization using genomic DNA from healthy mice leucocytes. For quantification, another standard curve (1 × 10^6^ to 1 × 10^0^) was prepared from the DNA of a culture of promastigote *L. infantum*.

Each DNA sample was used in two qPCR reactions. The first one targeted the *L. infantum* kDNA for parasite quantification and included 10 µL of GoTaq^®^ 2x qPCR Master Mix (Promega, Singapore), 10 pmol of each primer (kDNA5-F: 5′ CTTTTCTGGTCCTCCGGGTAGG 3′ and kDNA5-R: 5′ CCACCCGGCCCTATTTTACACCAA 3′) [20], and 100 ng of DNA with water to a final volume of 20 µL per reaction. The qPCR was performed into a thermocycler (Esco Swift, Esco, Singapore) using the following program: 10 min at 94 °C (1 cycle); 20 s at 94 °C, 20 s at 57 °C, and 20 s at 72 °C, with fluorescent acquisition at this last step (40 cycles). For sample normalization, another reaction was performed using the same amount of DNA and qPCR conditions but using primers for the mouse’s ubiquitin gene (Ubc) (UBC-F: 5′ AGGTCAAACAGGAAGACAGACGTA 3′ e UBC-R: 5′ TCACACCCAAGAACAAGCACA 3′) under an annealing temperature of 60 °C. All reactions were performed under three technical replicates and submitted to melting curve analysis after the amplification. The parasite burden of each sample was normalized to 106 mouse cells by the following formula [21]:

Quantity of L. infantum into the sampleNumber of Ubc copies into the sample2×106

### 2.6. Statistical Analysis

The results are presented as mean ± standard error of the mean (SEM). Statistical analysis was conducted using GraphPad Prism version 6.0 software (San Diego, CA, USA). The assumption of normality was evaluated by the Kolmogorov–Smirnov test. One-way ANOVA with multiple comparisons, followed by Tukey–Kramer post-test was used for analysis of the parasite burden. Two-way ANOVA with Bonferroni post-test was used for analysis of the immune response. A significance threshold of *p* < 0.05 was applied to all analyses.

### 2.7. Ethical Statements

All the experiments were conducted following the guidelines of the State University of Ceará Institutional Animal Care and Use Committee (protocol 3630450/2015).

## 3. Results

### 3.1. Antibody Production Kinetics

**Figure 1:**
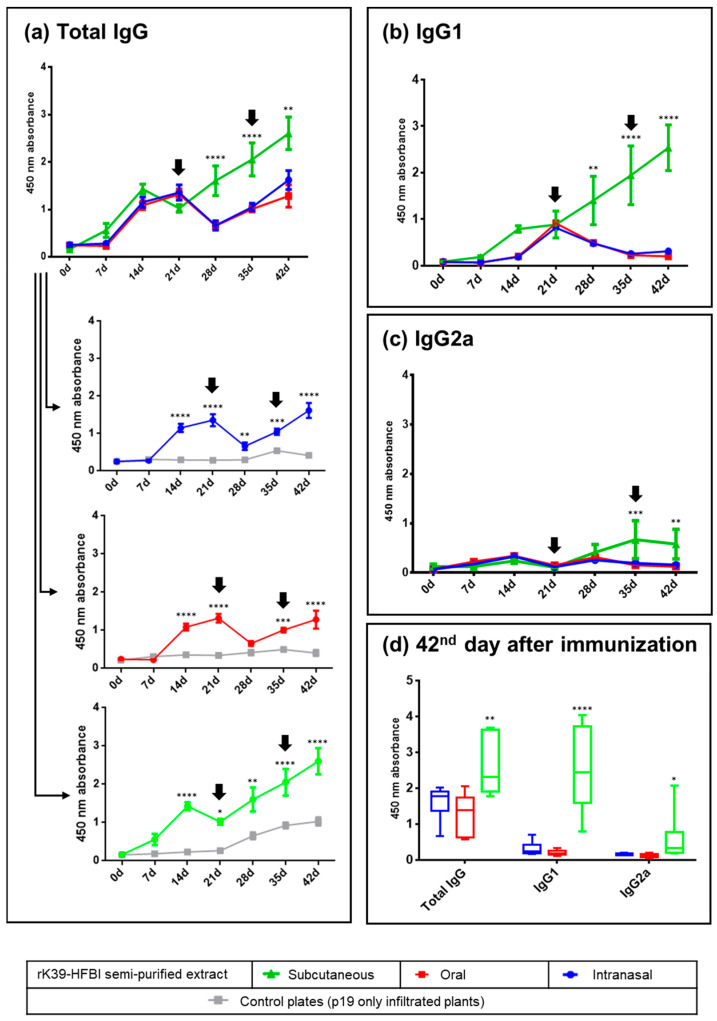
Kinetics of the rK39-HFBI-specific IgG, IgG1, and IgG2a production in BALB/c mice immunized by the intranasal, oral, and subcutaneous routes measured by ELISA. (**a**) IgG production of the three immunized groups, compared between each other (grouped graph) or against the control extract (individual graphs). (**b**) IgG1 and (**c**) IgG2a production elicited by the three immunization routes. (**d**) IgG, IgG1, and IgG2a production of all three groups at the time of the challenge with the parasite (42nd day). A serum dilution of 1:1000 was used. The antigen was semi-purified rK39-HFBI (blue, red, and green graphs) or semi-purified control plant extract (gray graphs). Arrows indicate the days of the booster doses. Mean ± SEM of each group is plotted. There was no statistically significant difference between the intranasal and oral groups in any analysis. The letters indicate statistical significance against all the other groups in the same graph and at the indicated time point (two-way ANOVA with Bonferroni post-test): * (*p* ≤ 0.05), ** (*p* < 0.01), *** (*p* < 0.001), and **** (*p* < 0.0001).

The present study demonstrated a systemic immune response in mice immunized with rK39-HFBI protein by the intranasal, oral, and subcutaneous routes. This response resulted in the synthesis of specific polyclonal antibodies (Figure 1).

All immunization regimens induced a primary immune response resulting in increased synthesis of IgG immunoglobulins on days 14 and 21 after immunization (Figure 1a). Following the booster, the subcutaneous group showed increased antibody synthesis as a secondary response on days 28, 35, and 42. In contrast, a decrease in antibody synthesis after the booster was observed in the oral and intranasal immunization groups. However, this effect was transient. This was evidenced by increased IgG levels on days 35 and 42. The specific immune response elicited by the semi-purified rK39-HFBI in all immunization routes was underlined by the low reactivity of the sera against the control plant proteins.

A transition from IgG isotypes to the IgG1 subclass was observed prior to the initial booster dose in all groups (Figure 1b). In the subcutaneous group, the titers of IgG1 continued to steadily increase over the course of 42 days. Conversely, both the intranasal and oral groups experienced a decrease in IgG1 titers following the first booster. The immunological response was maintained for both groups when the second booster dose was given on the 35th day.

The increase in the IgG2a subclass was observed only in the subcutaneous group as a secondary immune response (Figure 1c). At the time of the parasite challenge (42nd day), the subcutaneous group maintained elevated antibody titers compared to the mucosal groups, with a notable prevalence of the IgG1 subclass (Figure 1d). The production of specific antibodies against rK39-HFBI by oral immunization demonstrates the antigen’s stability in the highly denaturing environment of the digestive tract. Notably, in both mucosal surfaces, the first booster dose of the immunogen initially led to a reduction in antibody titers on the 28th day (Figure 1a). However, on the 35th day, the antibody levels of the intranasal and oral groups started to rise again, and this increase persisted after the second booster dose, suggesting that the initial suppression was only transient.

### 3.2. Parasite Burden

**Figure 2:**
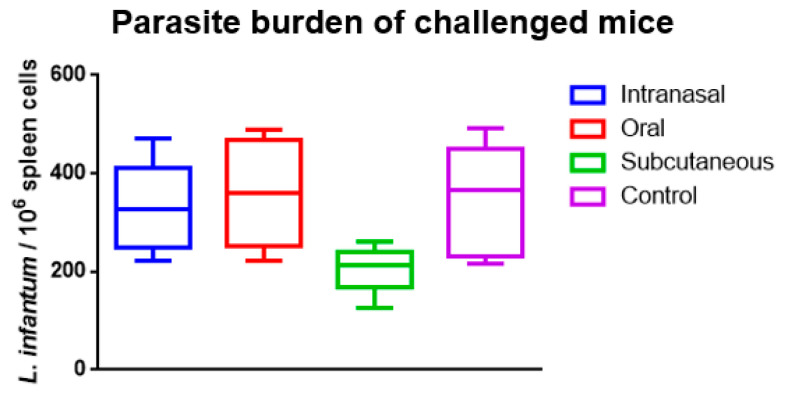
Splenic parasite burden over the rK39-HFBI immunized and control groups on the 42nd day after the challenge with *L. infantum*. The Mean ± SEM of each group is plotted. There was no statistical significance when analyzed by one-way ANOVA with multiple comparisons, followed by Tukey–Kramer post-test (*p* < 0.05).

On the 42nd day after the challenge (the 84th day after the first immunization), the splenic parasite burden of all groups was analyzed (Figure 2). The intranasal and oral groups were virtually identical to the control group. In contrast, there was an apparent decrease in the parasite burden in the subcutaneous group, although there was no statistical significance in this result.

## 4. Discussion

The production of a semi-purified fraction of the rK39-HFBI construct allowed the assessment of the immune response elicited by the K39 protein. The rK39-HFBI construct induced a systemic immune response in mice immunized by the subcutaneous, oral, and intranasal routes. The subcutaneous route was associated with increased production of IgG1 and IgG2a 42 days after the immunization. Upon challenge with the parasite, the results suggested a reduction in the parasite burden on the subcutaneous group, although no statistical difference could be found between the groups.

In mice, the antibody class switch to IgG1 or IgG2a is a T-helper (Th) cell-dependent event [22]. Extracellular antigens, such as the rK39-HFBI immunogen, usually induce Th2-type responses, which promotes class switching to IgG1 antibodies in response to interleukin-4 production. Thus, the high levels of IgG1 in the subcutaneous group may indicate a Th2-type response. However, additional markers should be assessed to confirm this finding. In addition, the IgG2a subclass was detected in the subcutaneous group after the boosters. This result suggests that a secondary immune response to the immunogen may be present in these animals. Quantification of other markers, including gamma interferon production, should be performed to confirm this finding [23].

Translation initiation factors eIF2 (LieIF2) and eIF2B (LieIF2B) are two intracellular antigens whose immunogenicity has also been characterized in *L. infantum*-infected BALB/c mice [24]. LieIF2B α, β, and γ subunits and LieIF2 α and β subunits induced mixed antibody responses, with immunoglobulins of both IgG1 and IgG2a subclasses detected, similar to what we reported for the rK39-HFBI antigen. On the other hand, the gamma subunit of LieIF2 induced predominantly IgG1 production. These results highlight the need for characterization studies of different antigens to better understand the immunological aspects of visceral leishmaniasis.

Pereira et al. [25] characterized the humoral immune response induced by recombinant repetitive antigens from the protozoan *Trypanosoma cruzi* in male BALB/c mice. According to these authors, while the Flagellar Repetitive Antigen (FRA) only induced IgG1 antibodies, the Cytoplasmic Repetitive Antigen (CRA) was able to induce a potent IgG1 and IgG3 response, as well as IgG2a and IgG2b, to a lesser extent. These findings support the specificity of the host’s effector response to the type of antigenic sequence presented, even in the presence of a genetic background that favors a Th2-type response by the host, such as the BALB/c strain [26].

In the present study, we also investigated whether prior immunization with the rK39-HFBI construct could influence parasite burden in animals challenged with *L. infantum* promastigote cells. However, there was no reduction in splenic parasite burden.

Other *L. infantum* intracellular antigens could also induce humoral immune responses in experimental models, with variable results regarding protection against visceral leishmaniasis. Immunization with the recombinant nucleosomal histones (HIS) and acidic ribosomal protein P0 (LiP0) were able to induce the production of high levels of specific antibodies, as reported by Pereira et al. [27]. However, compared to a DNA vaccine based on the same antigens, immunization with recombinant proteins showed poorer performance in protecting against a lethal dose challenge with *L. infantum*. Similar to our findings, these results reflect the activation of different immune pathways as a function of the site of antigen delivery. As a DNA vaccine, these antigens are expressed within the intracellular environment and presented by MHC-I molecules, resulting in a predominantly cellular immune response that is more effective in protecting the host [28].

A promising strategy for protection against visceral leishmaniasis is the induction of systemic immune responses by antigens delivered to mucosal surfaces [29,30]. The major drawbacks of these immunization routes are the harsh gastrointestinal environment, which could lead to antigen degradation, and the possibility of inducing immunological tolerance [31,32]. The rK39-HFBI construct elicited a systemic humoral immune response through both intranasal and oral routes, with significant production of total IgG. However, the low levels of IgG1 and IgG2a isotypes in these animals raise the possibility that these routes may have elicited other unassessed IgG isotypes. Consistent with our findings, other studies support the feasibility of using mucosal surfaces to elicit a systemic immune response, reinforcing their potential in the evaluation of new vaccines. Helou et al. [30] report that the intranasal administration of *L. donovani* antigens was more effective than the intradermal route in inducing protection against visceral leishmaniasis in female BALB/c mice. Furthermore, the use of *L. tarentolae* antigens administered as a mucosal vaccine via the rectal route was able to activate a Th1 immune response profile, while the same antigen administered subcutaneously induced a Th2 response [29].

This brief report represents the first report of the humoral immune response induced by the K39 antigen of *L. infantum*, demonstrating its high immunogenicity via the subcutaneous, oral, and intranasal routes. The main limitations of the present study include:

1. The use of a semi-purified fraction of the antigen produced in plants;
2. The use of the K39 antigen fused to the partner protein HFBI;
3. The use of *Quillaja saponaria* saponins as an adjuvant.

The serological analysis of the experimental groups against host plant proteins helped to reduce the immune response bias elicited by these contaminants. Although our protocol simulated the expected scenario for producing and using immunogens derived from a plant system [33], further analysis based on the purified antigen is still necessary for its complete characterization.

The fusion of the HFBI protein to the K39 antigen can be another confounding factor when analyzing the immune response to the rK39-HFBI construct. HFBI is a class II hydrophobin in the fungus *Trichoderma reesei* and is involved in developing hyphae. When fused to a partner protein, HFBI has been shown to facilitate the accumulation of the fused construct as protein bodies inside the plant cell and enable non-chromatographic purification of the protein of interest [34,35]. The immunogenicity of HFBI remains a subject of debate [36,37], which may necessitate its removal for a more accurate analysis of the K39.

*Q. saponaria* saponins have been used as an adjuvant in experimental models of mucosal vaccines, effectively replacing more toxic adjuvants such as cholera toxin or heat-labile toxin from *Escherichia coli* [38]. In formulations of recombinant vaccines developed in plant platforms, their addition to transgenic plant extracts induces a robust immune response and the activation of memory cells [39,40]. However, the elicited immune response profile can change depending on the saponin concentration [41], highlighting the need for further studies with different doses of saponins and different kinds of adjuvants to characterize the K39 antigen fully.

## 5. Conclusions

This study provided evidence of the robust humoral response induced by the K39 antigen of *L. infantum*, expressly when incorporated into the rK39-HFBI construct. The subcutaneous administration route predominantly stimulated the production of IgG1 isotype antibodies. However, additional investigations are necessary to clarify the IgG isotypes elicited by the oral and intranasal routes. While anti-K39 antibodies serve as the primary targets for serological diagnosis of visceral leishmaniasis, their involvement in the immunopathological aspects of this disease, such as hypergammaglobulinemia, still needs to be explored.

## Figures and Tables

**Table 1:** Experimental groups.

Group/Immunization Route	Immunization Protocol				Parasite Challenge
	Immunogen	Adjuvant	Total Volume	Immunization Timing	
Intranasal (n = 6 mice)	5 µg of semi-purified rK39-HFBI	8 µg of saponin	12 µL (6 µL per nostril)	First immunization (Day 0) Booster doses (21st and 35th days)	Intravenous administration of 1 × 10^7^ *L. infantum* promastigote cells (49th day)
Oral (n = 6 mice)	10 µg of semi-purified rK39-HFBI	16 µg of saponin	25 µL		
Subcutaneous (n = 6 mice)	20 µg of semi-purified rK39-HFBI	16 µg of saponin	40 µL		
Control (n = 6 mice)	None				

## Data Availability

The data presented in this study are available on request from the corresponding author.
